# Supplementary material for: The ENGAGE study: a randomized trial optimizing uptake of germline cancer genetic services in childhood cancer survivors
Source: Lancet Reg Health Am. 2026 Feb 13;55:101375. doi: 10.1016/j.lana.2026.101375 (PMC13001169; doi:10.1016/j.lana.2026.101375)
Supplement: Supplementary Tables [file mmc1.pdf]

The ENGAGE Study: A Randomized Trial Optimizing Uptake of Germline Cancer Genetic Services in Childhood Cancer Survivors

Supplementary Materials

Table of Contents	Page
Supplementary Table 1. Demographics of Responders versus Non-Responders.	2
Supplementary Table 2. Unadjusted and adjusted odds ratios for factors associated with uptake of genetic services in both arms (N = 391).	3
Supplementary Table 3. Unadjusted and adjusted odds ratios for factors associated with pre-test genetic counseling in the remote arm (n = 262).	4
Supplementary Table 4. Unadjusted odds ratios for factors associated with genetic testing uptake in the remote arm (n = 262).	5
Supplementary Table 5. Unadjusted odds ratios for factors associated with pre-test genetic counseling in the usual care arm (n = 129).	6
Supplementary Table 6. Unadjusted odds ratios for factors associated with genetic testing uptake in the usual care arm (n = 129).	7
Supplementary Table 7. Patients reported barriers to genetic services in both arms (n = 160).	8

Supplementary Table 1. Demographics of Responders versus Non-Responders.

	Consented (N = 391)	Refused/No Baseline Survey/ineligible post-consented (N = 164)	*p* - value
**Age at Invite**			0·054
Mean (SD)	43·97 (9·66)	45·86 (10·96)	
Median (Q1, Q3)	43·0 (37·0, 51·0)	46·0 (37·0, 55·0)	
Min, Max	24·0, 71·0	23·0, 69·0	
**Sex·**			0·66
Male	181 (46·3%)	80 (48·8%)	
Female	210 (53·7%)	84 (51·2%)	
**Race**			0·12
White	313 (80·1%)	122 (74·4%)	
Black	43 (11·0%)	31 (18·9%)	
American Indian/Alaskan Native	3 (0·8%)	0 (0·0%)	
Asian or Pacific Islander	18 (4·6%)	5 (3·0%)	
Other	12 (3·1%)	4 (2·4%)	
Unknown	2 (0·5%)	2 (1·2%)	
**Hispanic**			0·77
Yes	17 (4·3%)	5 (3·0%)	
No	360 (92·1%)	153 (93·3%)	
Unknown	14 (3·6%)	6 (3·7%)	
**ENGAGE refusal reason**			
Not Comfortable with Technology	0 (0·0%)	8 (4·9%)	
Not Interested	0 (0·0%)	61 (37·2%)	
Other	0 (0·0%)	53 (32·3%)	
Privacy/Security Concerns	0 (0·0%)	6 (3·7%)	
Too Busy	0 (0·0%)	12 (7·3%)	
Did not complete the baseline survey	0 (0·0%)	20 (12·2%)	
Missing information	0 (0·0%)	4 (2·4%)	

Supplementary Table 2. Baseline factors associated with uptake of genetic services in both arms (N = 391). Two Likert measures were reverse coded to facilitate interpretation.

		Did not have genetic services	Did have genetic services				
**Factor***	N	Mean (SD) or N (%)	Mean (SD) or N (%)	Unadjusted Odds Ratio (95%CI)	Unadjusted *p*-value	Adjusted Odds Ratio (95%CI)	Adjusted *p*-value
Usual care study arm (versus remote)	391	Remote 149 (57·5%)<br>Usual Care 110 (42·5%)	Remote 113 (85·6%)<br>Usual Care 19 (14·4%)	4·38 (2·49, 8·01)	<0·0001	4·53 (2·52, 8·14)	<0·0001
Genetic knowledge (per point increase in knowledge)	390	7·96 (3·49)	8·94 (3·21)	1·09 (1·02, 1·16)	0·0075	1·06 (0·98, 1·14)	0·15
Depression (per point increase in depression)	388	7·28 (3·77)	6·28 (2·78)	0·91 (0·85, 0·98)	0·0067	0·93 (0·84, 1·04)	0·21
Higher perceived odds of getting cancer again (per ordinal Likert increase)	389	3·65 (0·96)	3·86 (0·86)	1·29 (1·01, 1·66)	0·038	1·32 (1, 1·75)	0·050
Number FDR/SDR with cancer	391	1·54 (1·36)	1·70 (1·44)	1·08 (0·93, 1·26)	0·32	1·02 (0·85, 1·21)	0·86
Live in an urban area (vs. suburban/rural combined)	387	Suburban/Rural 203 (79·3%)<br>Urban 53 (20·7%)	92 (70·2%)<br>39 (29·8%)	1·62 (0·97, 2·70)	0·065	1·46 (0·83, 2·56)	0·19
Positive attitude toward genetic testing (per point increase)	390	29·50 (4·32)	31·20 (4·47)	1·09 (1·04, 1·15)	0·0003	1·05 (0·99, 1·12)	0·12
Not having a high deductible plan (relative to having one)	372	Deductible at least $1400: 92 (37·6%)<br>Deductible <$1400: 92 (37·6%)<br>Don't know: 61 (24·9%)	40 (31·5%)<br>67 (52·8%)<br>20 (15·7%)	1·67 (1·00, 2·81)	0·049	1·58 (0·91, 2·75)	0·10
Getting genetic testing feels complicated (per Likert increase)	390	2·61 (0·96)	2·36 (1·02)	Reverse coded OR 1·31 (1·05, 1·64)	0·017	1·04 (0·78, 1·38)	0·80
Genetic testing will cost me too much money (per ordinal Likert increase)	390	3·15 (0·84)	2·85 (0·90)	Reverse coded OR 1·51 (1·17, 1·96)	0·0014	1·17 (0·85, 1·62)	0·33
Anxiety (per point increase in anxiety)	387	8·07 (3·47)	7·31 (3·02)	0·93 (0·87, 1·00)	0·036	0·98 (0·88, 1·09)	0·67
Higher education attainment (per ordinal increase)	390	No college: 32 (12·4%)<br>Some college/Trade school: 74 (28·7%)<br>College+: 152 (58·9%)	10 (7·6%)<br>26 (19·7%)<br>96 (72·7%)	1·54 (1·09, 2·20)	0·012	1·31 (0·9, 1·9)	0·17
Age at invite	390	43·77 (9·61)	44·36 (9·79)	1·01 (0·98, 1·03)	0·58	1·00 (0·97, 1·02)	0·73
Self-Efficacy	390	15·69 (3·52)	16·13 (3·05)	1·04 (0·98, 1·11)	0·23	0·96 (0·89, 1·04)	0·34
Male (versus female)	391	Male 116 (44·8%)<br>Female 143 (55·2%)	65 (49·2%)<br>67 (50·8%)	1·19 (0·77, 1·85)	0·47	1·08 (0·65, 1·79)	0·77

SD=Standard deviation; N=Number of participants

Supplementary Table 3. Baseline factors associated with pre-test genetic counseling in the remote arm (n = 262).

		**Did not have pre-test genetic counseling**	**Did have pre-test genetic counseling**				
**Factor***	N	Mean (SD) or N (%)	Mean (SD) or N (%)	Unadjusted Odds Ratio (95%CI)	Unadjusted *p*-value	Adjusted Odds Ratio (95%CI)	Adjusted *p*-value
Genetic knowledge (per point increase in knowledge)	261	7·86 (3·68)	8·90 (3·16)	1·09 (1·02, 1·17)	**0·017**	1·06 (0·97, 1·16)	0·18
Depression (per point increase in depression)	259	7·60 (3·77)	6·24 (2·72)	0·88 (0·81, 0·95)	**0·0013**	0·94 (0·83, 1·06)	0·32
Higher perceived odds of getting cancer again (per ordinal Likert increase)	261	3·63 (1·02)	3·80 (0·85)	1·22 (0·93, 1·61)	0·161	1·22 (0·9, 1·67)	0·21
Number FDR/SDRs with cancer	262	1·56 (1·40)	1·66 (1·43)	1·05 (0·88, 1·25)	0·621	0·99 (0·81, 1·22)	0·94
Live in an urban area (vs. suburban/rural combined)	260	Suburban/Rural 121 (80·7%)<br>Urban 29 (19·3%)	75 (68·2%)<br>35 (31·8%)	1·94 (1·06, 3·59)	**0·031**	1·75 (0·92, 3·33)	0·087
Positive attitude toward genetic testing (per point increase)	261	29·23 (4·24)	31·11 (4·49)	1·10 (1·04, 1·17)	**0·0007**	1·05 (0·98, 1·13)	0·19
Not having a high deductible plan (relative to having one)	247	Deductible at least $1400: 54 (38·3%)<br>Deductible <$1400: 50 (35·5%)<br>Don't know: 37 (26.2%)	33 (31.1%)<br>54 (50.9%)<br>24 (17.9%)	1·76 (0·95, 3·29)	0·073	1·65 (0·88, 3·09)	0·12
Getting genetic testing feels complicated (per Likert increase)	261	2·57 (0·96)	2·34 (0·98)	Reverse coded<br>1·27 (0·98, 1·66)	0·075	1·08 (0·77, 1·52)	0·66
Genetic testing will cost me too much money (per ordinal Likert increase)	261	3·13 (0·85)	2·84 (0·91)	Reverse Coded<br>1·46 (1·09, 1·98)	**0·011**	1·06 (0·73, 1·54)	0·76
Anxiety (per point increase in anxiety)	259	8·28 (3·53)	7·24 (2·96)	0·91 (0·84, 0·98)	**0·013**	0·96 (0·85, 1·08)	0·48
Higher education attainment (per ordinal increase)	262	No College: 20 (13·2%)<br>Some College/Trade school: 42 (27·8%)<br>College+ 89 (58·9%)	10 (9·0%)<br>15 (13·5%)<br>86 (77·5%)	1·66 (1·12, 2·52)	**0·010**	1·45 (0·94, 2·24)	0·097
Age at Invite	262	43·85 (9·69)	44·23 (9·68)	1·00 (0·98, 1·03)	0·76	1·00 (0·97, 1·03)	0·90
Self-Efficacy	261	15·45 (3·80)	16·29 (3·06)	1·07 (1·00, 1·15)	0·058	0·99 (0·91, 1·09)	0·88
Male (versus female)	262	Male 64 (42·4%)<br>Female 87 (57·6%)	56 (50·5%)<br>55 (49·5%)	1·39 (0·82, 2·33)	0·24	1·15 (0·65, 2·04)	0·63

SD=Standard deviation; N=Number of participants

Supplementary Table 4. Baseline factors associated with genetic testing uptake in the remote arm (n = 262).

		**Did not have genetic testing**	**Had genetic testing**		
**Factor***	N	Mean (SD)	Mean (SD)	Unadjusted Odds Ratio (95%CI)	*Unadjusted p-value*
Genetic knowledge (per point increase in knowledge)	261	8·06 (3·57)	9·35 (3·02)	1·12 (1·02, 1·23)	**0·019**
Depression (per point increase in depression)	259	7·18 (3·57)	6·37 (2·65)	0·93 (0·83, 1·02)	0·14
Higher perceived odds of getting cancer again (per ordinal Likert increase)	261	3·64 (0·99)	3·96 (0·76)	1·48 (1·02, 2·22)	**0·038**
Number FDR/SDRs with cancer	262	1·61 (1·39)	1·59 (1·50)	0·99 (0·78, 1·24)	1.0
Live in an urban area (vs. suburban/rural combined)	260	Suburban/rural 164 (77·4%)<br>Urban 48 (22·6%)	32 (33·3%)<br>16 (33·3%)	1·70 (0·80, 3·52)	0·18
Positive attitude toward genetic testing (per point increase)	261	29·64 (4·37)	31·73 (4·38)	1·12 (1·04, 1·20)	**0·0028**
Not having a high deductible plan (relative to having one)	247	Deductible at least $1400: 73 (36·7%)<br>Deductible <$1400: 79 (39.7%)<br>Don't know: 47 (23·6%)	14 (29·2%)<br>25 (52·1%)<br>9 (18·8%)	1·65 (0·76, 3·71)	0·24
Getting genetic testing feels complicated (per Likert increase)	261	2·51 (0·98)	2·29 (0·91)	Reverse coded<br>1·28 (0·91, 1·80)	0·16
Genetic testing will cost me too much money (per ordinal Likert increase)	261	3·07 (0·88)	2·71 (0·87)	Reverse Coded<br>1·59 (1·09, 2·33)	**0·014**
Anxiety (per point increase in anxiety)	259	7·98 (3·43)	7·20 (2·84)	0·93 (0·84, 1·02)	0·15
Higher education attainment (per ordinal increase)	262	No college: 25 (11·7%)<br>Some college/trade school: 47 (22·1%)<br>College+ 141 (66·2%)	5 (10·2%)<br>10 (20·4%)<br>34 (69·4%)	1·11 (0·69, 1·85)	0·77
Age at Invite	262	44·27 (9·80)	42·88 (9·11)	0·98 (0·95, 1·02)	0·37
Self-Efficacy	261	15·66 (3·62)	16·43 (3·00)	1·07 (0·97, 1·18)	0·17
Male (versus female)	262	Male 94 (44·1%)<br>Female 119 (55·9%)	26 (53·1%)<br>23 (46·9%)	1·43 (0·73, 2·78)	0·33

SD=Standard deviation; N=Number of participants

Supplementary Table 5. Baseline factors' association with pre-test genetic counseling in the usual care arm (n = 129).

	Had Genetic Counseling in Usual Care Arm				
	No	Yes	Total		
Variable	N = 110<br>Mean (SD)	N = 19<br>Mean (SD)	N = 129<br>Mean (SD)	Odds Ratio (95% CI)	Unadjusted p-value
Genetic knowledge (per point increase in knowledge)	8·14 (3·21)	9·13 (3·67)	8·29 (3·29)	1·10 (0·94, 1·30)	0·22
Depression (per point increase in depression)	6·89 (3·76)	6·21 (2·94)	6·79 (3·65)	0·94 (0·80, 1·09)	0·49
Higher perceived odds of getting cancer again (per ordinal Likert increase)	3·69 (0·87)	4·11 (0·88)	3·75 (0·88)	1·88 (0.96, 3·95)	0·068
Number FDR/SDRs with cancer	1·49 (1·32)	2·11 (1·41)	1·58 (1·34)	1·35 (0·95, 1·92)	0·091
Live in an urban area (vs. suburban/rural combined)	Suburban/rural 83 (76·9%)<br>Urban 25 (23·1%)	16 (84.2%)<br>3 (15·8%)	28 (22·0%)	0·62 (0·11, 2·45)	0·71
Positive attitude toward genetic testing (per point increase)	29·83 (4·38)	32·13 (4·39)	30·17 (4·44)	1·14 (1·01, 1·30)	0·033
Not having a high deductible plan (relative to having one)					0.40
Yes	39 (36·8%)	6 (31·6%)	45 (36·0%)		
No	43 (40·6%)	12 (63·2%)	55 (44·0%)	1·80 (0·56, 6·45)	
Don't know	24 (22·6%)	1 (5·3%)	25 (20·0%)		
Getting genetic testing feels complicated (per Likert increase)	2.67 (0.96)	2.42 (1.30)	2.64 (1.02)	Reverse coded<br>1·28 (0·77, 2·17)	0·38
Genetic testing will cost me too much money (per ordinal Likert increase)	3.18 (0.81)	2.89 (0.94)	3.14 (0.84)	Reverse coded<br>1·52 (0·81, 2·93)	0·22
Anxiety (per point increase in depression)	7·82 (3·35)	7·47 (3·42)	7·77 (3·35)	0·97 (0·83, 1·12)	0·72
Higher education attainment (per ordinal increase)				0·99 (0·46, 2·32)	1·0
No college	12 (11·0%)	0 (0·0%)	12 (9·4%)		
Some college/trade school	33 (30·3%)	10 (52·6%)	43 (33·6%)		
College+	64 (58·7%)	9 (47·4%)	73 (57·0%)		
Age at Invite	43.60 (9·49)	45.53 (10·89)	43.88 (9·69)	102 (0·97, 1·07)	0·43
Self-Efficacy	16.03 (3·05)	15.21 (3·05)	15.91 (3·05)	0·92 (0·79, 1·08)	0·30
Male (versus Female)	50 (49·%)<br>56 (50·9%)	7 (36·8%)<br>12 (63·2%)	61 (47·3%)<br>68 (52·7%)	0·61 (0·19, 1·82)	0·46

Supplementary Table 6. Baseline factors' association with pre-test genetic testing in the usual care arm (n = 129).

	Had Genetic Testing in the Usual Care arm				
	No	Yes	Total		
	N = 117<br>Mean (SD)	N = 12<br>Mean SD	N = 129<br>Mean SD	Odds Ratio (95% CI)	Unadjusted p-value
Genetic knowledge (per point increase in knowledge)	8·27 (3·20)	8·46 (4·23)	8·29 (3·29)	1·02 (0·85, 1·23)	0·87
Depression (per point increase in depression)	6·84 (3·70)	6·33 (3·20)	6·79 (3·65)	0·96 (0·78, 1·13)	0·72
Higher perceived odds of getting cancer again (per ordinal Likert increase)	3·68 (0·87)	4·42 (0·67)	3·75 (0·88)	3·72 (1·40, 11·71)	0·0047
Number FDR/SDRs with cancer	1·54 (1·31)	2·00 (1·65)	1·58 (1·34)	1·26 (0·81, 1·89)	0·31
Live in an urban area (vs. suburban/rural combined)	Suburban/Rural<br>88 (76·5%)<br>Urban 27 (23·5%)	11 (91·7%)<br>1·00 (8·3%)	28 (22·0%)	0·30 (0·01, 2·23)	0·41
Positive attitude toward genetic testing (per point increase)	29·82 (4·36)	33·50 (4·03)	30·17 (4·44)	1·26 (1·07, 1·53)	0·0044
Not having a high deductible plan (relative to having one)					
Yes	40 (35·4%)	5 (41·7%)	45 (36·0%)		
No	49 (43·4%)	6 (50·0%)	55 (44·0%)	0·98 (0·23, 4·38)	1·00
Don't know	24 (21.2%)	1 (8.3%)	25 (20.0%)		
Getting genetic testing feels complicated (per Likert increase)	2.68 (1.01)	2.25 (1.06)	2.64 (1.02)	Reverse Coded<br>1·53 (0·81, 3·02)	0·22
Genetic testing will cost me too much money (per ordinal Likert increase)	3.15 (0.83)	3.08 (0.90)	3.14 (0.84)	Reverse Coded<br>1·09 (0·51, 2·38)	0·95
Anxiety (per point increase in depression)	7·83 (3·38)	7·17 (3·10)	7·77 (3·35)	0·94 (0·76, 1·13)	0·56
Higher education attainment (per ordinal increase)				1·06 (0·41, 3·21)	1.0
No college	12 (10·3%)	0 (0·0%)	12 (9·4%)		
Some college/trade school	37 (31·9%)	6 (50·0%)	43 (33·6%)		
College+	67 (57·8%)	6 (50·0%)	73 (57·0%)		
Age at Invite	43.75 (9·52)	45.17 (11·55)	43.88 (9·69)	1·02 (0·95, 1·08)	0·64
Self-Efficacy	15.91 (3·08)	15.83 (2·92)	15.91 (3·05)	0·99 (0·82, 1·22)	0·95
Female (versus male)	56 (47·9%)<br>61 (52·1%)	5 (41.7%)<br>7 (58·3%)	61 (47.3%)<br>68 (52·7%)	0·78 (0·18, 3·03)	0·92

Supplementary Table 7. Patients reported barriers to genetic services in both arms (n = 160).

Coded themes and examples of patient quotes	Overall (N=160)	Remote Genetic Services (n=90)	Usual Care (n=70)
**Time constraints, No. (%)**<br>"*I've been busy working full-time for the first time.*"<br>*"Busy with family."*<br>*"Could not get around to it, but will attempt to in the near future."*	45 (28·1)	22 (24·0)	23 (32·9)
**Not enough information regarding the process or rationale. No. (%)**<br>*"Wasn't clear on how, who, and where to do this. It is too difficult for me to understand and process, possibly due to my chemo brain."*<br>*"I don't know. That's not something I've ever been advised to do."*<br>*"Didn't know what they were talking about."*<br>*Confusion about the process itself primarily, cost secondary."*	32 (20%)	18 (20·0)	14 (18·7)
**Low perceived value, no interest, No. (%)**<br>*"Don't feel I need it."*<br>*"Diagnosis is already confirmed. No need for genetic counseling at this time."*<br>*"Not necessary, as I don't have any health issues."*	29 (18·1)	16 (17·8)	13 (18·6)
**Concerns about cost or insurance coverage, No. (%)**<br>"*I also cannot afford to pay for anything right now. I have no extra funds to spend on testing/doctor appointments. Unfortunately, we're using every penny we've got just to get by."*<br>*"I don't think it would be affordable. Insurance companies are reluctant to cover anything these days."*<br>*"I also had to do all the legwork at the expense of the testing, call my insurance company, and work with the clinic. It took about half a day to figure out if it would be covered. Even as I went for my appointment, I wasn't 100% sure if the test itself would be covered. I feel like this was a stressor."*	26 (16·3)	10 (9%)	16 (22·8)
**Poor follow-through or difficult scheduling, No. (%)**<br>*"Thought they were supposed to call me automatically.:*<br>*"I forgot to schedule."*	18 (11·3)	18 (20)	0
**Hard to access, No. (%)**<br>"*Small town…I don't know how or where to receive testing."*<br>*"No computer at home."*<br>*"I reached out to my provider (they don't do that) and a place I found online without response."*<br>*"I don't drive and live far from the hospital to get it done. Also, my family doctor said they do not do it there, so that was not an option. I was told I would need to go see a specialist, and I just cannot do that. It was too difficult to get done."*	13 (8·1)	5 (5·5)	8 (10·7)
**Not a priority, No. (%)**<br>*"I was in the middle of a job search, and it was not a priority."*<br>*"One of my children was diagnosed with Autism, and I have been super busy trying to get things situated with him."*<br>*"Have let myself become distracted with other responsibilities and have ignored my personal health care interests."*	7 (4·4)	7 (7·7)	0
**Provider didn't support, No. (%)**<br>*"I asked my provider, but they deemed it unnecessary and could not provide a referral or recommendation."*	4 (2·5)	0	4 (5·7)

Other less frequently reported barriers were anxiety (n=1 in usual care), discomfort in sharing personal information (n=1 in remote services), and planning to test later (n=2 in remote services). Some said they might test in the future.
